# Supplementary material for: A spatial memory signal shows that the parietal cortex has access to a craniotopic representation of space
Source: eLife. 2018 Feb 16;7:e30762. doi: 10.7554/eLife.30762 (PMC5834243; doi:10.7554/eLife.30762)
Supplement: Transparent reporting form [file elife-30762-transrepform.pdf]

eLIFE
1st Floor
24 Hills Road
Cambridge CB2 1JP, UK
P 01223 855340
W elifesciences.org
T @elife

eLife's transparent reporting form

We encourage authors to provide detailed information *within their submission* to facilitate the interpretation and replication of experiments. Authors can upload supporting documentation to indicate the use of appropriate reporting guidelines for health-related research (see EQUATOR Network), life science research (see the BioSharing Information Resource), or the ARRIVE guidelines for reporting work involving animal research. Where applicable, authors should refer to any relevant reporting standards documents in this form.

If you have any questions, please consult our Journal Policies and/or contact us: editorial@elifesciences.org.

Sample-size estimation

- You should state whether an appropriate sample size was computed when the study was being designed
- You should state the statistical method of sample size computation and any required assumptions
- If no explicit power analysis was used, you should describe how you decided what sample (replicate) size (number) to use

Please outline where this information can be found within the submission (e.g., sections or figure legends), or explain why this information doesn't apply to your submission:

We determined the sample size using 95% confidence interval (z-score of 1.96) for our true population and the right statistical power. Most of our analysis resulted in really low p-values (< 0.0001). Overall, we followed what is generally accepted as reasonable, as depicted in this Matlab script:

```
Zscore = 1.96;%confidence
stdv = 0.5; % expected variance
MErr = 0.05;  %+/- 5 CI
Need_Sample_size = round((Zscore^2 * (stdv*(1-stdv)))/ MErr^2);
```

Replicates

- You should report how often each experiment was performed
- You should include a definition of biological versus technical replication
- The data obtained should be provided and sufficient information should be provided to indicate the number of independent biological and/or technical replicates
- If you encountered any outliers, you should describe how these were handled
- Criteria for exclusion/inclusion of data should be clearly stated
- High-throughput sequence data should be uploaded before submission, with a private link for reviewers provided (these are available from both GEO and ArrayExpress)

Please outline where this information can be found within the submission (e.g., sections or figure legends), or explain why this information doesn't apply to your submission:

We studied 288 LIP cells in 3 monkeys. Two of the monkeys were studied by one author (SCS) and the other, with the larger sample, was studied by the other author (MS). Because we used single-electrode recording, the experiments were done over several years. The results were similar in all three monkeys. All neurons in our sample had visual responses to the onset of a saccade target in their receptive fields, and exhibited delay-period and/or presaccadic activity in the memory-guided delayed saccade task. Furthermore, their postsaccadic responses were not contaminated by the phasic and tonic components of an eye position signal often found in parietal neurons (Andersen et al., 1998). We included every neuron with those characteristics in our sample.

Statistical reporting

- Statistical analysis methods should be described and justified
- Raw data should be presented in figures whenever informative to do so (typically when N per group is less than 10)
- For each experiment, you should identify the statistical tests used, exact values of N, definitions of center, methods of multiple test correction, and dispersion and precision measures (e.g., mean, median, SD, SEM, confidence intervals; and, for the major substantive results, a measure of effect size (e.g., Pearson's r, Cohen's d)
- Report exact p-values wherever possible alongside the summary statistics and 95% confidence intervals. These should be reported for all key questions and not only when the p-value is less than 0.05.

Please outline where this information can be found within the submission (e.g., sections or figure legends), or explain why this information doesn't apply to your submission:

The methods section has detailed description of statistical methods used and each figure, its corresponding explanation text and legend, have specific information on the particulars of what is experimentally found and depicted.

(For large datasets, or papers with a very large number of statistical tests, you may upload a single table file with tests, Ns, etc., with reference to sections in the manuscript.)

Group allocation

- Indicate how samples were allocated into experimental groups (in the case of clinical studies, please specify allocation to treatment method); if randomization was used, please also state if restricted randomization was applied
- Indicate if masking was used during group allocation, data collection and/or data analysis

Please outline where this information can be found within the submission (e.g., sections or figure legends), or explain why this information doesn't apply to your submission:

Neurons were grouped using monkeys, tasks and their visual and memory responses.

Additional data files ("source data")

- We encourage you to upload relevant additional data files, such as numerical data that are represented as a graph in a figure, or as a summary table

eLife Sciences Publications, Ltd is a limited liability non-profit non-stock corporation incorporated in the State of Delaware, USA, with company number 5030732, and is registered in the UK with company number FC030576 and branch number BR015634 at the address 1st Floor, 24 Hills Road, Cambridge CB2 1JP | August 2014

- Where provided, these should be in the most useful format, and they can be uploaded as "Source data" files linked to a main figure or table
- Include model definition files including the full list of parameters used
- Include code used for data analysis (e.g., R, MatLab)
- Avoid stating that data files are "available upon request"

Please indicate the figures or tables for which source data files have been provided:

We have uploaded all Matlab codes used in the data analysis. Some commented lines are left as such to show they have been used as alternative approaches. We also have uploaded an excel file with the numerical data which includes all the analysis results. The file has headers (column names) for the particular parameter calculated. Careful naming approach has been used to be precise and concise. For instance, pMemoBaseMAX represents ranksum p-value for memory vs baseline maximum firing rates (trial comparisons, for the memory window considered: -125 ms to 500 ms around saccade start)
